# Reproductive health for refugees by refugees in Guinea III: maternal health

**DOI:** 10.1186/1752-1505-5-5

**Published:** 2011-04-12

**Authors:** Natasha Howard, Aniek Woodward, Yaya Souare, Sarah Kollie, David Blankhart, Anna von Roenne, Matthias Borchert

**Affiliations:** 1 London School of Hygiene and Tropical Medicine (LSHTM), Keppel Street, London, UK; 2 Reproductive Health Group (RHG), Guéckédou, Guinea; 3 Gesellschaft für Technische Zusammenarbeit (GTZ) GmbH, 65726 Eschborn, Germany; 4 Institute of Tropical Medicine and International Health, Charité-Universitätsmedizin Berlin, Germany; 5 Institute of Tropical Medicine, Antwerp Belgium

## Abstract

**Background:**

Maternal mortality can be particularly high in conflict and chronic emergency settings, partly due to inaccessible maternal care. This paper examines associations of refugee-led health education, formal education, age, and parity on maternal knowledge, attitudes, and practices among reproductive-age women in refugee camps in Guinea.

**Methods:**

Data comes from a 1999 cross-sectional survey of 444 female refugees in 23 camps. Associations of reported maternal health outcomes with exposure to health education (exposed versus unexposed), formal education (none versus some), age (adolescent versus adult), or parity (nulliparous, parous, grand multiparous), were analysed using logistic regression.

**Results:**

No significant differences were found in maternal knowledge or attitudes. Virtually all respondents said pregnant women should attend antenatal care and knew the importance of tetanus vaccination. Most recognised abdominal pain (75%) and headaches (24%) as maternal danger signs and recommended facility attendance for danger signs. Most had last delivered at a facility (67%), mainly for safety reasons (99%). Higher odds of facility delivery were found for those exposed to RHG health education (adjusted odds ratio 2.03, 95%CI 1.23-3.01), formally educated (adjusted OR 1.93, 95%CI 1.05-3.92), or grand multipara (adjusted OR 2.13, 95%CI 1.21-3.75). Main reasons for delivering at home were distance to a facility (94%) and privacy (55%).

**Conclusions:**

Refugee-led maternal health education appeared to increase facility delivery for these refugee women. Improved knowledge of danger signs and the importance of skilled birth attendance, while vital, may be less important in chronic emergency settings than improving facility access where quality of care is acceptable.

## Background

Three-quarters of maternal deaths occur during delivery or the immediate post-partum period [1]. An estimated 358,000 women worldwide died from pregnancy-related causes in 2008, commonly from preventable or treatable conditions such as haemorrhage, eclampsia, obstructed labour, sepsis, and unsafe abortion [2-4]. The maternal mortality ratio (MMR) globally has decreased 1-3% annually since 1990, but this will not achieve Millennium Development Goal (MDG) 5 - to improve maternal health - for which an annual decline of 5.5% is needed. In Sub-Saharan Africa, where the annual decline remains 0.1%, improved maternal knowledge and access to care is considered vital in saving women's lives [3,5]. Skilled attendance at birth is a key global intervention in reducing maternal mortality [6].

Conflict and displacement are associated with poverty, loss of livelihood, disruption of services, breakdown of social support systems, and increased sexual violence, and are generally accompanied by reduced capacity to respond to reproductive health needs, further complicating provision of maternal care [2,3,5-10]. Maternal and neonatal mortality among refugees can be high [7]. A study of Afghan refugees in Pakistan showed 41% of deaths among reproductive-age women were pregnancy-related, due to inaccessibility of emergency obstetric care. Studies on refugee maternal health in developing countries are still relatively rare. This study enabled insight into the influences of refugee-led health education, formal schooling, parity, and age on maternal knowledge, attitudes and practices among reproductive-age refugee women in Guinea.

### Setting

Fifteen years of conflict in Liberia and Sierra Leone displaced over 500,000 people into the Forest Region of neighbouring Guinea [11]. Many Liberians returned home after 1997 elections, while the Sierra Leone conflict lasted until 2002. Two major refugee influxes in the early and late nineties strained Guinean health services, weakened by governmental economic policies. Guinea's Ministry of Health integrated refugee health services into the health system. Refugees received free care at Guinean facilities, costs covered by the United Nations High Commissioner for Refugees (UNHCR). However, antenatal care attendance was only 11-42% for refugees, while almost 100% for Guineans, with some refugees reporting government reproductive health services as unsatisfactory [12]. In 2008, MMR was 860 per 100,000 live births for women living in Guinea, 859 in Liberia, and 1,033 in Sierra Leone with the latter being one of the highest recorded in the world [3].

### Programme

A full description of the programme and services provided is published in von Roenne et al [12]. In 1995, a group of refugee midwives and laywomen supported by German Technical Cooperation (GTZ) established the non-governmental organisation '*Reproductive Health Group' *(RHG). Aiming to improve services for fellow refugees in Guéckédou and Kissidougou prefectures, RHG recruited refugee nurses and midwives to local Guinean health facilities and trained refugee laywomen to provide reproductive health education, referrals, and contraceptives for their communities [12].

As part of developing and strengthening programming, RHG staff conducted operational research when stability and funding allowed. Data for this study was collected during a 1999 cross-sectional reproductive health interview survey of refugees in the Forest Region [13].

### Objectives

The primary objective was to assess whether exposure to RHG facilitator-led health education was associated with differences in maternal knowledge, attitudes, or practices. Secondary objectives were to assess whether age, parity or education, were associated with differences in maternal knowledge, attitudes or practices.

## Methods

### Study design

Methodology was published in detail elsewhere [13]. Maternal healthcare as used here focuses on the continuum of care during antenatal, natal, and postnatal periods [8]. The target population was female refugees of reproductive age (15 to 49) from an estimated population of 125,000 women living in 48 camps across Guinea's Forest Region where RHG had been active for four years. Sampling was multi-stage. First, 45 clusters of households were randomly selected in 23 camps, with probability of selection proportional to camp size. Second, a stratified sample of ten women per cluster was randomly selected from household lists. Sample size was calculated to detect a difference of 10% versus 20% between strata of equal size with 80% power and 95% confidence level (95%CI), accounting for clustering. Participation was voluntary, with no reimbursement beyond travel costs. Ethical approval was provided by the Ministry of Public Health in Guinea and the London School of Hygiene & Tropical Medicine (LSHTM) in the UK.

### Data collection and analysis

The questionnaire was adapted from those used and validated in similar developing-country settings and piloted in a camp excluded from the study [13]. Additional questions were added relevant to specific RHG maternal health education content. To improve reliability, questions were read verbatim in English, the language used by most respondents, and only translated or rephrased if a respondent did not understand. Prompting was only used for certain questions where multiple answers were possible (e.g. danger signs for pregnant women). Female interviewers were recruited from the refugee community, trained for four days, and given instruction on issues including privacy, prompting, and translations. Data was double-entered in Epi-Info™6, with range and consistency checks to reduce transposition error [13,14].

Analysis was conducted using Stata^®^11. Associations of maternal health variables with exposure to RHG facilitators, parity, education level, and age, were analysed using logistic regression.

The study assessed maternal knowledge, attitudes and practices of women on topics previously taught through RHG activities. Exposure to RHG-led health education was categorised as *exposed *if participants reported their main source of family planning (FP) information as an RHG facilitator or drama group and *unexposed *if not. Women receiving family-planning advice also received pregnancy-related information. Authors also used arrival at camp before or after 1996 as a comparative proxy, as all participants who had been in camp prior to 1996 could be assumed to have been exposed to RHG activities [13].

Formal educational attainment was categorised as *some *(any primary education or more) or *none *(no formal education). Education was selected as it is a social determinant of health, positively affecting knowledge, social skills, and discussion about health, all of which better equip women to access and use health information and services [15]. Women with some formal education could be expected to have improved knowledge, attitudes and practices compared to women without any formal education.

Age was categorised as *adolescent *(15-19) or *adult *(20-49). Age was explored because adolescents have specific reproductive needs that are often not as well-addressed as those of women 20 years and above [16]. For example, young mothers' physical immaturity heightens their risk of mortality or morbidity from obstructed labour, fistula, and premature birth [17].

Parity was categorised as *nulliparous*, *parous *or *grand multiparous *(having delivered five or more infants), the last being considered a risk factor in subsequent pregnancies. Parity was explored because it seemed logical that women who have given birth would have increased maternal knowledge and possibly different attitudes and practices. Previous research in this population showed parity had a significant association with FP knowledge, indicating it might have a significant association with general reproductive health knowledge and practices [13].

Period of arrival in camp was categorised as *pre-1996 *or *post-1995 *to account for different waves of migration.

Location of most recent delivery was categorised as *home *(i.e. with or without skilled assistance) or *facility *(e.g. delivery at a hospital, health post, or health centre with skilled assistance). Home deliveries typically took place without the assistance of a skilled birth attendant [6].

O*bstetric need *was defined as having experienced penetrative sex and not currently abstaining or using any modern family planning method, as this could lead to pregnancy and the need for maternal healthcare.

Clustering was accounted for using robust standard errors. Potential confounders, including RHG exposure, age, formal education, arrival period in camp, religion, and marriage age, were selected according to published literature on maternal health and refugees and expert discussion. To maintain the strength of multivariate models, potential confounders (except marriage age and religion) were coded as binary after determining that this did not alter odds ratios (ORs). Confounders were retained in multivariate models if they changed odds ratios by at least 10%.

## Results

### Demographics

The response rate exceeded 95% and the total sample was 444 women. Table 1 shows most respondents were from Sierra Leone (97%) and had arrived in camp after 1995 (58%). Only 29% had received some formal education. Almost all (94%) were sexually experienced. Most (72%) were married, 74% during adolescence, and 32% reported their husband as having multiple wives. The majority of women were parous (81%), including 31% grand-multiparous, and had an average of two children living with them. Approximately 69% of women last delivered at a facility, while approximately 68% would potentially need obstetric care in the next twelve months.

**Table 1:** Demographic characteristics

Variable	Women (%)
*All respondents:*	*n = 444 (100)*

**Age**	
15-24 (Adolescent)	190 (43)
25-47 (Adult)	254 (57)
	
**Country of origin**	
Sierra Leone	432 (97)
Liberia	12 (3)
	
**Arrival in camp**	
Before 1996	188 (42)
1996 or later	256 (58)
	
**Education**	
No formal education	316 (71)
Some formal education	128 (29)
	
**Religion**	
Catholic	88 (20)
Protestant	184 (41)
Muslim	172 (39)
	
**Age at first penetrative sex**	
15 years or less	228 (51)
16 years or older	185 (42)
Unknown	5 (1)
Never	26 (6)
	
**Marital status**	
Never married	69 (16)
Currently married	320 (72)
Widowed/Separated	55 (12)
	
**Parity**	
Nulliparous	84 (19)
Primiparous	64 (14)
Multiparous (2-4)	159 (36)
Grand multipara (5-14)	137 (31)
	
Obstetric need*	
No - Never had penetrative sex	26 (6)
No - Current FP user/abstaining	115 (26)
Yes	303 (68)

*Ever married respondents*	*n = 375 (100)*

**Partner has other wife/partners**	120 (32)
	
**Currently living with partner**	275 (73)
	
**Age at marriage**	
10 or under	12 (4)
11-14	39 (10)
15-17	226 (60)
18-29	96 (26)
30+	1 (0)

*Parous female respondents*	*n = 360 (100)*

**Living children**	
None	36 (10)
1-3 children living in household	258 (72)
4-8 children living in household	66 (18)
	
**Place of last delivery**	
At home	111 (31)
At facility	249 (69)

**NB: **No prompting was used. *Obstetric need covers all women who have had sex and do not currently use any family planning.

### Exposure to RHG activities

Table 2 shows the association of RHG exposure with maternal knowledge, attitudes, and practices. No significant differences were found in maternal knowledge or attitudes between RHG-exposed and unexposed women. Almost all respondents (99%) said women should attend antenatal care (ANC), primarily for safe pregnancy and delivery (81%). Most respondents reported abdominal pain (75%) or headaches (24%) as danger signs, while other signs including vaginal bleeding and oedema were never mentioned. Despite not reporting several danger signs, 96% of women said they would seek facility care if they considered themselves at risk.

**Table 2:** Maternal health knowledge, attitudes, and practices, comparing women exposed to RHG health education to those unexposed

Variable	Unexposed (%)	RHG-exposed (%)	OR^c ^(95%CI)
*All respondents:*	*n = 171 (100)*	*n = 273 (100)*	

Pregnant women should attend ANC	168 (98)	273 (100)	..

*Respondents who agreed women should attend ANC:*	*n = 168 (100)*	*n = 273 (100)*	..

**Main reason to attend ANC** (unprompted answer)			
Safe pregnancy/delivery^d,e,g^	137 (81)	219 (80)	0.76 (0.41-1.42)
Healthy children^d,e,g^	15 (9)	34 (13)	1.88 (0.85-4.17)
Vaccination/Other^d,e,g^	16 (10)	20 (7)	0.84 (0.38-1.88)

**Reasons for vaccination in pregnancy**			
To protect against tetanus^d,e,g^	156 (91)	264 (97)	2.22 (0.52-9.43)

*All respondents:*	*n = 171 (100)*	*n = 273 (100)*	

Danger signs in pregnancy*,**			
Headaches^d-g^	40 (23)	72 (26)	1.17 (0.70-1.97)
Abdominal pain^d-g^	131 (77)	201 (74)	0.85 (0.51-1.43)
Vaginal bleeding/Oedema	0 (0)	0 (0)	..
**Actions if danger signs present**			
Visit health facility	160 (94)	269 (99)	
Other/Don't know^d,g^	11 (6)	4 (1)	4.32 (0.79-23.61)

*Parous respondents*	*n = 134 (100)*	*n = 225 (100)*	

**Place of last delivery**			
Facility	81 (60)	168 (74)	
Home ^a,d-g^	53 (40)	57^b ^(26)	1.93 (1.23-3.01)

*If last delivered at facility*	*n = 81 (100)*	*n = 168 (100)*	

Reasons for facility delivery*,**			
Safety^e,f^	80 (99)	166 (99)	1.32 (0.11-15.67)
Staff competence ^a,d,e,g^	75 (94)	140 (82)	0.37 (0.15-0.90)
Staff attitude^d-g^	65 (80)	138 (82)	1.13 (0.57-2.25)
Privacy^d-g^	65 (81)	120 (71)	0.58 (0.29-1.14)
Referred by health staff^d-g^	38 (48)	98 (58)	1.55 (0.82-2.93)
Nearness of facility^d-g^	44 (54)	86 (51)	0.91 (0.54-1.51)
Costs ^a,d,e,g^	14 (18)	55 (33)	2.50 (1.34-4.69)

*If last delivered at home:*	*n = 53 (100)*	*n = 57^b ^(100)*	

Reasons for home delivery*,**			
Distance to facility^g^	48 (91)	55 (96)	4.97 (0.79-31.15)
Privacy^g^	30 (57)	30 (53)	0.61 (0.26-1.44)
Staff competence^d,e,g^	19 (36)	20 (35)	0.93 (0.41-2.13)
Costs^d,g^	14 (26)	21 (36)	1.27 (0.51-3.20)
Staff attitude ^a,g^	18 (34)	14 (25)	0.37 (0.17-0.83)
Tradition^e,g^	7 (13)	9 (16)	1.30 (0.55-3.08)

NB: *prompting may have been used; ** multiple answers possible; ªSignificant p-value (p ≤ 0.05); ^b ^One participant was removed from analysis as she did not report where she delivered; ^c ^Adjusted for age, education, period of arrival (pre/post 1996), age at marriage; ^d ^Adjusted OR excludes education; ^e ^Adjusted OR excludes period of arrival; ^f ^Adjusted OR excludes age at marriage; ^g ^Adjusted OR excludes age.

RHG-exposed women had almost twice the odds of unexposed women of having last delivered in a facility (OR 1.93; 95%CI 1.23-3.01). Safety (99%) and staff competence (88%) were the main reasons reported for choosing facility delivery. RHG-exposed women were 63% less likely to report staff competence as reason for facility deliver (OR 0.37, adjusted for age at marriage; 95%CI 0.15-0.90), but had 2.5 times higher odds of reporting cost as reason for facility delivery (OR 2.50, adjusted for age at marriage; 95%CI 1.34-4.69). The main reasons reported for home delivery by all respondents were distance to a facility (94%) and privacy (55%). Large camps (e.g. over 10,000 population) had dedicated health centres, while smaller camps shared centres. However, camp size was not associated with choice of home delivery or with perceived distance to health facilities. Using period of arrival as proxy for RHG exposure provided similar results.

### Education and age

Having any formal education was not associated with maternal health knowledge or attitudes. Educated women had almost twice the odds of last having delivered at a facility (OR 1.93, adjusted for age at marriage; 95%CI 0.96-3.92), though this was not significant. Safety (99%) and staff competence (87%) were the main reasons reported for facility delivery, while distance to facility (97%) and privacy (50%) were the main reasons given for home delivery.

No significant differences were found in maternal knowledge, attitudes, or practices among adolescents versus mature women.

### Parity

No significant differences were found between nulliparous and parous women regarding main reasons for attending ANC, vaccinations, or recognition of danger signs. Parous women recognised abdominal pain (77% versus 64%), while nulliparous women recognised headaches (36% versus 23%) more frequently (OR 1.86, adjusted for age, education, arrival period; 95%CI 1.09-3.17). Approximately 95% of women said they would go to a facility if experiencing danger signs.

Table 3 compares grand multiparous (≥5 births) and lower parity (1-4 births) women by place of last delivery and reasons given. Grand multiparous women had almost twice the odds of having last delivered at a facility compared with lower-parity respondents (OR 1.85, adjusted for marriage age; 95%CI 1.06-3.23). Safety (98%) and distance to facility (94%) remained the main reasons for facility or home delivery respectively, with no significant differences by parity.

**Table 3:** Place of last delivery and reasons, comparing grand multiparous (≥5 births) to parous women (1-4 births)

Variable	Parous (%)	G. Multipara (%)	OR^c ^(95%CI)
*All parous respondents:*	*n = 223 (100)*	*n = 136^b ^(100)*	

**Place of last delivery**			
Facility	144 (65)	105 (77)	
Home^a, d,e,g^	79 (35)	31 (23)	1.85 (1.06-3.23)

*If last delivered at facility:*	*n = 144 (100)*	*n = 105 (100)*	

Reasons for facility delivery*,**			
Safety^e,f^	143 (99)	103 (98)	0.78 (0.11-5.37)
Staff competence^d,e,f^	124 (86)	91 (88)	1.25 (0.49-3.19)
Staff attitude^d-h^	118 (82)	85 (81)	0.94 (0.48-1.83)
Privacy^d-f,h^	113 (79)	72 (69)	0.54 (0.27-1.07)
Referred by health staff^d-f^	79 (55)	57 (54)	0.97 (0.55-1.74)
Nearness of facility^d-f,h^	79 (55)	51 (48)	0.56 (0.31-1.04)
Costs^d-f^	43 (30)	26 (25)	0.56 (0.28-1.12)

*If last delivered at home:*	*n = 79 (100)*	*n = 31 (100)*	

Reasons for home delivery*,**			
Distance to facility^e,f^	74 (94)	29 (94)	0.80 (0.11-5.83)
Privacy^d-f,h^	44 (56)	16 (52)	0.56 (0.18-1.75)
Staff competence^d,e,h^	25 (32)	14 (45)	1.51 (0.49-4.67)
Costs^d-f,h^	23 (29)	12 (39)	1.06 (0.43-2.60)
Staff attitude	24 (30)	8 (26)	0.81 (0.25-2.61)
Tradition^e,h^	7 (9)	9 (29)	4.01 (0.80-20.15)

NB: *prompting may have been used; ** multiple answers possible; ªSignificant p-value (p ≤ 0.05); ^b ^One participant was removed from analysis as she did not report where she delivered; ^c ^Adjusted for age, education, period of arrival, age at marriage, RHG exposure; ^d ^Adjusted OR excludes education; ^e ^Adjusted OR excludes period of arrival; ^f ^Adjusted OR excludes age at marriage; ^g ^Adjusted OR excludes age; ^h ^Adjusted OR excludes RHG exposure.

## Discussion

This study indicates that the majority of participants (68%) had potential obstetric need. According to the literature, 15% of these women would require emergency obstetric care [18]. While access to basic and comprehensive emergency obstetric care was not measured, 68% potential need indicates the importance of maternal support and access to care for these refugee women. The high obstetric need (Table 1) appeared related to low levels of contraceptive usage, largely due to desire for more children. Young women did not appear to have greater difficulties than mature women in accessing services. Research by the authors and others has indicated that refugee demand drove much of the improvements in government health services [12,19].

Maternal knowledge levels were generally low and did not differ significantly by exposure to RHG activities. This did not seem due to the exposure proxy measure used, as time in camp provided similar results. Reasons for this are unclear as maternal health education was provided in RHG sessions. Lack of maternal health knowledge can negatively affect access to needed care [20]. Increasing maternal knowledge among refugee women, especially recognition of danger signs beyond abdominal pain and headaches, could improve care seeking and thus birth outcomes [6,21,22]. A study of Afghan refugees showed that providing information on danger signs in pregnancy increased timely seeking of skilled birth support [23]. A possible reason for better knowledge transmission in the Afghan study was the higher number of educational workers serving a smaller population, with 330 volunteer community health workers and 325 female health workers for 96,300 male and female refugees (1:300) versus 75 facilitators for 125,000 female refugees (1:1,700) in Guinea [12,23]. This suggests that observable increases in maternal knowledge and attitudes require considerable staff investment. However, it is also possible that RHG sessions emphasised family planning or STIs rather than maternal health, or that key content was missed, as health education quality was not included in this assessment.

Neither formal education, nor age, nor parity was significantly associated with maternal knowledge or attitudes. These findings reinforce earlier findings from this population that age and education were only weakly associated with sexually-transmitted disease or family planning knowledge and practice outcomes [13,14]. Two potential explanations present themselves. First, in traditional African settings adolescent women are not necessarily informed about maternal health, potentially gaining knowledge with each pregnancy and ANC visit. For example, Benner et al show that young women are often unaware they could become pregnant during first sex [24]. Second, in this population ANC attendance covered approximately 54% of expected deliveries and data was not available on numbers of ANC visits [12]. Thus, while almost all respondents said pregnant women should attend ANC, many did not do so and consequently missed ANC-delivered health education and support. Research shows a positive association between ANC attendance and facility delivery, with women who attend at least four times most likely to deliver at facilities [25,26]. Low ANC attendance may have reduced observable age and parity differences [12-16,27].

While research suggests that skilled birth attendance is most frequently sought for first deliveries, with care-seeking decreasing as parity increases, this study suggests the reverse [6]. Grand multiparas, along with formally-educated women, were significantly more likely than others to have delivered most recently at a facility. It is possible that previous negative experiences during conflict or as refugees increased risk-aversion, as safety was the main reason reported for facility delivery. As responses were adjusted for child mortality, fear of personal harm appeared the main reason. Findings indicate that despite a lack of maternal health knowledge, most women chose delivery options they considered safer. More research in refugee settings is needed to determine possible reasons and why this differs from other research.

Age and education were not significantly associated with place of last delivery, unlike other research showing older or less-educated women as less likely to have skilled birth attendance [6,25,28]. It is possible that refugee status reduced traditional family-based coping mechanisms, causing women to choose safer professional deliveries when possible. Additionally, refugees may have had access to better facilities than were available in Sierra Leone, though this would contradict refugee reports of poorer reproductive health services in Guinea [12].

While safety was the main reason women reported for choosing facility delivery, distance was one of the main reasons they did not. This suggests that while many women preferred facility delivery, poor accessibility was a barrier. Cost was the most significant difference between RHG-exposed and unexposed women in choosing facility delivery, also indicating that poor affordability (e.g. due to perceived costs, travel costs, under-table costs) might be a barrier. Facility delivery costs, approximately US$6 for a girl and US$7.5 for a boy (2009 United States dollar constants), were paid by UNHCR, while home-delivery costs were not. It is possible that RHG-exposed women were more aware of free services, thus favouring facility delivery.

Countries implementing the "skilled birth attendance in health facility" approach generally have significantly lower maternal mortality ratios than those that do not, depending upon the appropriateness, accessibility, and quality of care [29]. Poor-quality infrastructure, lack of transport, and population dispersal affect access to delivery services [20,30,31]. There were 28 health facilities, including the district hospital, within the refugee zone of Guéckédou and Kissidougou districts, with comprehensive emergency obstetric care available in each larger camp. Camp size was not associated with reporting of distance as a barrier to facility delivery in this population. More research could determine whether perceived or actual distances are a greater barrier to facility delivery. Skilled attendant coverage was approximately 24% in this population, showing significant improvement would be needed to reach the 90% coverage required to meet MDG 5 by 2015 [12,32].

The authors are confident of the representativeness of the sample, having minimised reporting and observer bias through training and piloting, and reduced Type I error (false positive results) through robust standard errors methods. Available confounders were addressed, though unmeasured confounding may exist as data on factors such as socio-economic status and gender-based violence was missing. Cross-sectional studies do not account for time-sequence and the authors do not attribute causality or disregard potential reverse causality. While plausible that exposure to RHG activities encouraged facility deliveries, authors cannot rule out that those who preferred facility delivery may also have sought family planning advice from RHG. Alternatively, women who opted for facility delivery because they were particularly risk-aware or perhaps wanted a 'modern' delivery might also attend RHG sessions, without those sessions affecting their choice of delivery location.

In categorising RHG exposure, authors assumed that those women unable to explain family planning, and therefore not asked about their main information sources for family planning, had not been exposed to RHG activities. As this was a potentially significant assumption, authors compared findings with those using period of arrival at camp as RHG-exposure proxy, as all participants in camp prior to 1996 would have been exposed to RHG activities. Findings were similar with both proxy exposure measures, indicating that assumptions were reasonable. Family-planning/drama session participation was considered a more valid indicator than period of arrival at camp as it relies on reported rather than proximal exposure.

Postnatal care coverage was only 12% of expected deliveries in this refugee population [12]. While RHG staff was aware that many women did not attend postnatal services, the reasons remain unclear as follow-up research could not be conducted. Postnatal care is often overlooked yet remains important for approximately 20 million women and babies affected by conflict and displacement and consequently the progression of MDG 5 [33].

Since the 1994 ICPD Conference in Cairo, there have been some positive changes in reproductive health in Guinea, particularly the increased rates of contraceptive usage [13,19]. Guinean reproductive health services did not reach refugee women in the Forest Region effectively before RHG began activities. Prior to the refugee influx, the Forest Region was much less populated, with health centres few and far between and a population not accustomed to reproductive health service coverage. Refugee demand for better reproductive health services and RHG support of Guinean nurses working in tandem with and learning from Liberian and Sierra Leonean nurses significantly improved services [12]. However, as most refugees have since returned to their countries of origin, RHG has stopped operating in Guinea. Meanwhile, Guinea has been suffering very difficult political and economic times and health services appear now in a worse state in this area than when RHG was active.

Despite a general lack of maternal health knowledge, most respondents said that ANC was important, that they would seek professional help for danger signs, and that they had last delivered in a facility (i.e. sought skilled birth attendance). That women exposed to RHG health education had significantly higher odds of facility delivery suggests the positive effect of RHG activities on skilled birth attendance and thus maternal health [1-3]. Overall, authors are encouraged that exposure to RHG's 'maternal healthcare for refugees by refugees' is associated with higher prevalence of facility-based 'skilled' deliveries, but concerned that ANC attendance was low by African standards and refugees had significant knowledge gaps regarding maternal danger signs. More research is recommended to determine how accessibility to maternal health information and care in chronic conflict areas can be improved.

## Competing interests

The authors declare that they have no competing interests.

## Authors' contributions

NH and AW analysed the data and drafted the paper. NH gave final approval of the version for publication. DB, SK and YS contributed to conception and design, acquisition of data, and reviewing the paper. AvR conceived the study, and contributed to design, data interpretation, and reviewing the paper. MB designed the study, contributed to data acquisition and interpretation, and critically reviewed the paper. All authors approved the version to be published.

## References

[B1] Islam M The safe motherhood initiative and beyond Bulletin of the WHO 2007 85 Geneva: WHO 733 820 10.2471/BLT.07.045963 PMC2636492 18038048

[B2] Souza JP Maternal near miss and maternal death in the World Health Organisation's 2005 global survey on maternal and perinatal health Bulletin of the WHO 2010 88 113 119 10.2471/BLT.08.057828 PMC2814475 20428368

[B3] Hogan MC Foreman KJ Naghavi M Ahn SY Wang M Makela SM Lopez AD Lozano R Murray CJ Maternal mortality for 181 countries, 1980-2008: a systematic analysis of progress towards Millennium Development Goal 5 Lancet 2010 375 9726 1609 23 10.1016/S0140-6736(10)60518-1 20382417

[B4] WHO Trends in Maternal Mortality: 1990 to 2008 Geneva 2010

[B5] WHO Maternal Mortality in 2005 - Estimates Developed by WHO, UNICEF, UNFPA and the World Bank 2007 Geneva: WHO

[B6] Stanton C NLanc AK Croft T Choi J Skilled care at birth in the developing world: progress to date and strategies for expanding coverage 2006 39 Cambridge Journals 109 120 10.1017/S0021932006001271 16522226

[B7] Bartlett LA Jamieson DJ Kahn T Sultana M Wilson HG Duerr A Maternal mortality among Afghan refugees in Pakistan, 1999-2000 Lancet 2002 359 643 649 10.1016/S0140-6736(02)07808-X 11879858

[B8] Bryce J Daelmans B Dwivedi A Fauveau V Lawn JE Mason E Newby H Shankar A Starrs A Wardlaw T Countdown to 2015 for maternal, newborn, and child survival: the 2008 report on tracking coverage of interventions Lancet 2008 371 1247 1258 10.1016/S0140-6736(07)61694-8 18406859

[B9] Burnham G Maternal deaths among Afghan refugees Lancet 2002 359 639 640 10.1016/S0140-6736(02)07780-2 11879855

[B10] Msuya D Report on Assessment of Reproductive Health Services for Adolescent Refugees in Guinea's Forest Region WHO 1999

[B11] Van Damme W De Brouwere V Boelaert M Van Lerberghe W Effects of a refugee-assistance programme on host population in Guinea as measured by obstetric interventions Lancet 1998 351 1609 1613 10.1016/S0140-6736(97)10348-8 9620714

[B12] von Roenne A von Roenne F Kollie S Swaray Y Sondorp E Borchert M Reproductive health services for refugees by refugees: an example from Guinea Disasters 2010 34 16 29 10.1111/j.1467-7717.2009.01112.x 19459901

[B13] Howard N Kollie S Souare Y von Roenne A Blankhart D Newey C Chen MI Borchert M Reproductive health services for refugees by refugees in Guinea I: family planning Confl Health 2008 2 12 10.1186/1752-1505-2-12 18925936 PMC2579911

[B14] Chen MI von Roenne A Souare Y von Roenne F Ekirapa A Howard N Borchert M Reproductive health for refugees by refugees in Guinea II: sexually transmitted infections Confl Health 2008 2 14 10.1186/1752-1505-2-14 18947393 PMC2582230

[B15] Higgins C Lavin T Metcalfe O Health impacts of education: a review 2008 The Institute of Public Health in Ireland

[B16] Chynoweth SK The need for priority reproductive health services for displaced Iraqi women and girls Reprod Health Matters 2008 16 93 102 10.1016/S0968-8080(08)31348-2 18513611

[B17] USAID Maternal and child health - technical areas USAID http://www.usaid.gov/our_work/global_health/mch/mh/techareas/adolescent.html accessed 1 April 2011

[B18] Ronsmans C Campbell OM McDermott J Koblinsky M Questioning the indicators of need for obstetric care Bull World Health Organ 2002 80 317 324 12075369 PMC2567763

[B19] McGinn T Allen K Improving refugees' reproductive health through literacy in Guinea Glob Public Health 2006 1 229 248 10.1080/17441690600680002 19153909

[B20] Kottegoda S Samuel K Emmanuel S Reproductive health concerns in six conflict-affected areas of Sri Lanka Reprod Health Matters 2008 16 75 82 10.1016/S0968-8080(08)31359-7 18513609

[B21] Anya SE Hydara A Jaiteh LE Antenatal care in The Gambia: missed opportunity for information, education and communication BMC Pregnancy Childbirth 2008 8 9 10.1186/1471-2393-8-9 18325122 PMC2322944

[B22] Freedman LP Graham WJ Brazier E Smith JM Ensor T Fauveau V Themmen E Currie S Agarwal K Practical lessons from global safe motherhood initiatives: time for a new focus on implementation Lancet 2007 370 1383 1391 10.1016/S0140-6736(07)61581-5 17933654

[B23] Purdin S Khan T Saucier R Reducing maternal mortality among Afghan refugees in Pakistan Int J Gynaecol Obstet 2009 105 82 85 10.1016/j.ijgo.2008.12.021 19232603

[B24] Benner MT Townsend J Kaloi W Htwe K Naranichakul N Hunnangkul S Carrara VI Sondorp E Reproductive health and quality of life of young Burmese refugees in Thailand Confl Health 2010 4 5 10.1186/1752-1505-4-5 20338037 PMC2907534

[B25] Fotso JC Ezeh AC Essendi H Maternal health in resource-poor urban settings: how does women's autonomy influence the utilization of obstetric care services? Reprod Health 2009 6 9 10.1186/1742-4755-6-9 19531235 PMC2706794

[B26] Bosmans M Nasser D Khammash U Claeys P Temmerman M Palestinian women's sexual and reproductive health rights in a longstanding humanitarian crisis Reprod Health Matters 2008 16 103 111 10.1016/S0968-8080(08)31343-3 18513612

[B27] Drummond PD Mizan A Wright B HIV/AIDS knowledge and attitudes among West African immigrant women in Western Australia Sex Health 2008 5 251 259 10.1071/SH07077 18771640

[B28] Babalola S Fatusi A Determinants of use of maternal health services in Nigeria--looking beyond individual and household factors BMC Pregnancy Childbirth 2009 9 43 10.1186/1471-2393-9-43 19754941 PMC2754433

[B29] Campbell OM Graham WJ Strategies for reducing maternal mortality: getting on with what works Lancet 2006 368 1284 1299 10.1016/S0140-6736(06)69381-1 17027735

[B30] Wayte K Zwi AB Belton S Martins J Martins N Whelan A Kelly PM Conflict and development: challenges in responding to sexual and reproductive health needs in Timor-Leste Reprod Health Matters 2008 16 83 92 10.1016/S0968-8080(08)31355-X 18513610

[B31] Furuta M Mori R Factors affecting women's health-related behaviors and safe motherhood: a qualitative study from a refugee camp in eastern Sudan Health Care Women Int 2008 29 884 905 10.1080/07399330802269600 18726797

[B32] WHO Millennium Development Goal 5 Geneva 2008

[B33] UNHCR Statistical Yearbook Chapter II - Population levels and trends 2007

